# DNA characterization reveals potential operon-unit packaging of extracellular vesicle cargo from a gut bacterial symbiont

**DOI:** 10.21203/rs.3.rs-3689023/v1

**Published:** 2023-12-04

**Authors:** Byeongyeon Cho, Grace Moore, Loc-Duyen Pham, Chirag J. Patel, Aleksandar D. Kostic

**Affiliations:** Department of Biomedical Informatics, Harvard Medical School, Boston, MA, USA; Department of Biomedical Informatics, Harvard Medical School, Boston, MA, USA; Kostic Lab, Joslin Diabetes Center, Boston, MA, USA; Department of Biomedical Informatics, Harvard Medical School, Boston, MA, USA; Kostic Lab, Joslin Diabetes Center, Boston, MA, USA

## Abstract

Extracellular vesicles (EVs) are lipid bilayer-bound entities secreted by cells across all domains of life, known to contain a range of components, including protein complexes, RNA, and DNA. Recent studies on microbial extracellular vesicles indicate that these virus-sized nanoparticles, 40–90nm in diameter, readily cross the epithelial barrier and reach systemic circulation, can be detected in tissues throughout the body in mice and that 1mL of plasma from healthy humans contains up to one million bacterial EVs. They have been recently recognized for their biologically functional roles, including modulation of bacterial physiology and host-microbe interactions, hence their gain in the microbiome research community’s attention. However, the exact understanding of their functionality is still a subject of active research and debate. Here, we employ long-read DNA sequencing on purified extracellular vesicles from a common mammalian gut symbiont, *Parabacteroides goldsteinii*, to characterize the genomic component within EV cargos. Our findings challenge the notion of DNA packaging into EVs as a stochastic event. Instead, our data demonstrate that the DNA packaging is non-random. Here, we suggest a novel hypothesis of selective EV-DNA packaging, potentially arranged in operon units, hence providing new insights into our understanding of its genetic makeup and its potential role, underlining the importance of our findings in microbial community dynamics.

## Introduction

Organisms from all biological domains are capable of releasing extracellular vesicles ^1–4^. Bacterial extracellular vesicles (EVs) have traditionally been considered a cellular mechanism for expelling misfolded proteins and other unwanted substances^5^. Studies have demonstrated that these lipid bilayer-bound EVs contain RNA, DNA, lipid-based compounds, immunologically active protein complexes, and molecules. The composition and function of these EVs vary across different bacterial species ^6–11^. EVs are now recognized as more than mere protective microbial survival mechanisms for bacterial waste disposal. They are believed to play biologically functional roles depending on the specific cargo components. Both Gram negative and Gram positive bacteria have been found to release EVs ^11^, which influence bacterial physiology, host-microbe interactions, and horizontal gene transfer ^2,12,13^. It has also been reported that prokaryotic EVs can contain genes coding for resistance to antibiotics, virulence, and metabolic characteristics ^14^. Despite these discoveries, the rules governing the composition and the relative quantity of EV-DNA cargo across various bacterial species remain largely uncharacterized, emphasizing the need for further exploration in this field ^12,14^.

Recent studies have underscored the potential role of EVs in contributing to innate bacterial defense, particularly in its response to bacteriophage infection ^14^. Notably, phage-lysis-derived EVs are reported to contain more DNA than those from β-lactam antibiotic-induced blebbing, as seen in Staphylococcus aureus EVs, which has also been demonstrated to provide bacterial resistance against membrane-targeting antibiotic ex-vivo and in-vitro ^13^. Furthermore, variations in DNA cargo characteristics have been observed in EVs exposed to antibiotics in a phage-dependent manner ^15^. However, gaps remain in our comprehensive understanding of the EVs’ relationship with phages ^13^.

Recent work has identified the function of EVs in facilitating horizontal gene transfer (HGT), with evidence showing their ability to mediate gene exchange both within and across bacterial species, even extending to different genera ^13^. EVs have been shown to provide a safeguarded milieu for genetic material and facilitate intercellular gene transfer ^14^ and have been reported to contribute to bacterial genome evolution and potential antibiotic resistance gene transfer ^8,15^. Environmental stressors have also been shown to escalate EV production and gene transfer rates^14^. However, the mechanisms underlying EV biogenesis regulation, DNA cargo content’s relationship to HGT, and EV-recipient cell fusion remain incompletely understood ^4^.

Studies have uncovered the potential for selective protein and RNA packaging mechanisms. For instance, in *Bacteroides fragilis* and *Bacteroides thetaiotaomicron*, proteins in EVs and outer membranes are reported to be selectively packaged based on their acidic protein charge, a process with implications in nutrient acquisition ^14,16^. Additionally, the lipid composition in *Porphyromonas gingivalis* has been shown to influence protein selection in EVs ^17^. Concurrently, studies profiling RNA transcriptomes in secreted EVs further underscore the specificity of these packaging processes ^14,18^. However, the mechanisms of EV-DNA cargo selection and its role have been significantly understudied and have been gaining significant attention in recent years. For example, the EV-DNA cargos in *Streptomyces coelicolor* are shown to span the entire bacterial chromosome, raising questions about the functionality of this DNA and its packaging ^18^. Similarly, in *Pseudomonas aeruginosa* EVs, genome-wide DNA fragments have been reported, with an enrichment of regions linked to virulence and antibiotic resistance ^4,19^. Yet, the precise rules and mechanisms behind this selectivity remain poorly defined. The extent to which the DNA content in these vesicles results from random processes rather than selective packaging and how the genetic materials within membrane vesicles influence the host-microbe evolutionary processes, both within and between species, is a considerable unknown in the field ^14^.

This study investigates the genomic composition of extracellular vesicles from the Oxford Nanopore Technology long-read genomic DNA sequences of *P. goldsteinii* strain ASF519 ^20^. Our study assesses the randomness of the EV-DNA cargo content by examining whether a specific subset of genes consistently exhibits differential abundance compared to that of theoretical random gene packaging. Through a multifaceted analytical pipeline, we provide evidence against the randomness of DNA cargo loading. Additionally, we aim to determine if EV-DNA packaging is selective for specialized biological functions. Our study reveals that the genomic packaging of EVs is a non-random process and potentially functionally selective of specific gene sets. We also find that EVs are capable of packaging DNA from any region of the genome, which supports the findings of recent studies ^8,19^. Importantly, our study proposes a novel potential mechanism suggesting selective EV-DNA packaging. We demonstrate the grouped nature of DNA packaging into extracellular vesicles, suggesting a gene sorting mechanism of EVs is arranged in operon units. These results have important implications for understanding the role of EVs in biological processes.

## Results

### Ground truth control DNA count generation

**Extended Data Fig. 1:**
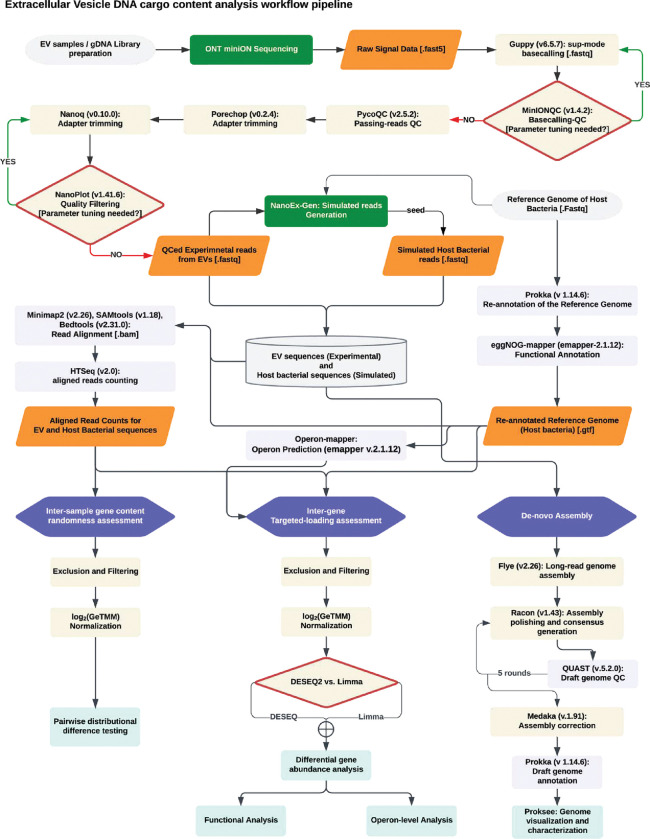
Bioinformatics pipeline architecture: study workflow diagram

To investigate whether bacterial EVs encapsulate a predominantly random selection of DNA fragments from their parent bacterial cell or whether they are selectively packaged, we developed an analytical pipeline suitable for statistical comparison of normalized gene counts using the long-read DNA sequencing data from EVs and the reference genome of this bacterial strain (Extended Data Fig. 1).

**Extended Data Fig. 2:**
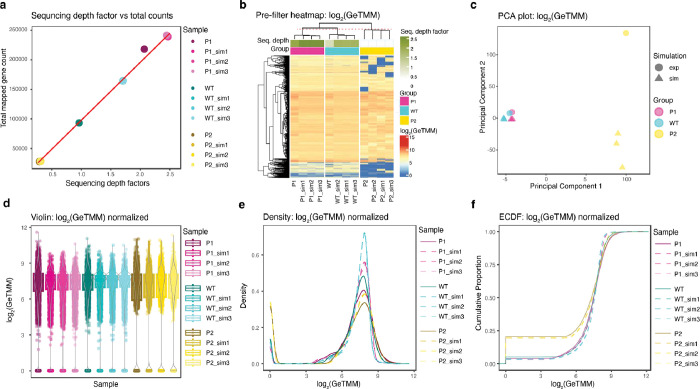
Deliberate inclusion of low-sequencing depth sample (P2) validate the suitability of the study’s control-group generation, filtering and normalization method (Supplementary information). Exploratory analysis (a-f) conducted on 5391 unfiltered mapped reads including the low-sequencing depth sample, P2, shows that (a) sequencing depth is a significant factor in the variability of gene counts. (b) Hierarchical clustering heatmap across all mapped reads (unfiltered) indicates the overall similarities within groups and higher variability induced by lower sequencing depth. The simulated control group for the P2 sample highlights regions that could induce false positives due to low-sequencing depth. (c) Principal component analysis shows EVs and parent bacterium representation within the same cluster, where principal component 1 is likely to explain variability introduced by sequencing depth while principal component 2 explains the biological differences. (d) Violin plot, (e) Density plot, and (f) Empirical cumulative density function demonstrate the reliability of the generated control sample closely mimicking the sequencing condition of each experimental sample and the suitability of the GeTMM normalization method in incorporating sequencing depth and gene length differences.

For a fair statistical comparison, the ground truth control group’s (parent bacterial cell) gene count set should accurately represent the theoretically expected mapped reads counts if the genetic material is uniformly distributed at random to EVs. The quantification of mapped read counts is probabilistically contingent upon factors including gene length, sequencing depth, and sequencing condition. Therefore, we perform comparisons between the theoretical genomic DNA (gDNA) sequencing data from the donor bacteria *P. goldsteinii* ASF519 produced by our long-reads simulator NanoEx-Gen and the experimental sequencing data derived from mapping EV sequencing reads to the ASF519 reference genome (Supplementary Notes). Three rounds of theoretical control group generating simulations were performed for each experimental group. We performed GeTMM normalization to account for both read length and sequencing depth variability in differential abundance analysis (Extended Data Fig. 2). The normalized gDNA copy counts of EVs from a transposon-insertion mutant (denoted P1) (insertion of “TTC GCT CTG TTC AAT A” at the OIDBDKIG_02333 locus) and Wild Type ASF519 (denoted WT) are compared to those of their theoretical simulated counts from donor bacteria.

### DNA copy count distribution of the EVs and its parent bacterium *P. goldsteinii* ASF519

**Figure 1:**
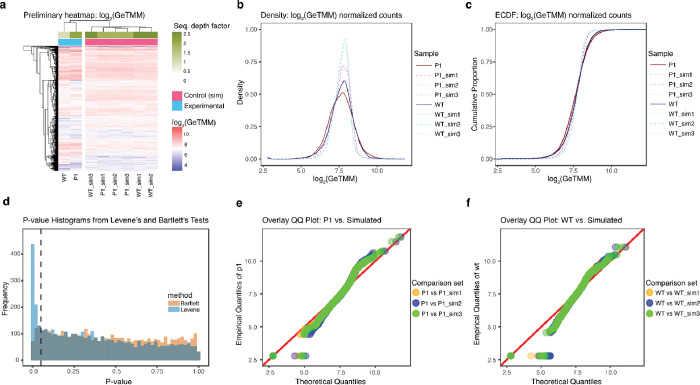
Central gene distribution of the EVs and its parent bacterium *P. goldsteinii* ASF519 is similar, but evidence shows that subsets of genes are differentially overrepresented or underrepresented in the EVs. (a) Hierarchical clustering heatmap across 4437 pre-filtered normalized gene counts shows the similarities between gene abundance patterns within control and experimental groups and the effect of sequencing depth difference in variability of counts. Normalized DNA counts distribution between EVs and their donors are shown using (b) density plot and (c) Empirical cumulative density function. (D) Levene’s and Bartlett’s unadjusted p-value histogram for equal variance testing between control and experimental groups, with a dashed line indicating the p-value of 0.05. Q-Q plots comparing the gene count distribution between EVs and their donor bacterium counterparts for (E) P1 samples and (F) WT samples.

The heatmap representation of the pre-filtered log_2_(GeTMM) normalized gene copy counts (Fig. 1a) illustrates a hierarchical clustering along the horizontal axis, distinctly grouping the samples from EVs (specifically P1 and WT) together while separately clustering all samples representative ASF519 parent bacterial group labeled with the suffix <_sim>. The observed amplified variability for lower-count genes is attributable to coverage-induced variability. The density plots (Fig. 1b) exhibit similarities among biological groups, as evidenced by closely aligned patterns and distances, suggesting a general similarity in gene copy count between EVs and their parent bacteria. The empirical cumulative density function (Fig. 1c) shows the overall distributional similarity of the DNA counts within the EV cargo and its donor. To ascertain if the variances across the experimental group (from EV) paralleled those in the simulated group (from donor bacteria) for both P1 and WT categories, we applied Bartlett’s ^21^ and Levene’s ^22^ tests. Levene’s and Bartlett’s unadjusted p-value histogram (Fig. 1d) shows non-significant evidence for equal variance between the groups, hence our choice for non-parametric methods for formal statistical testing of distributional similarities, encompassing the Mann-Whitney U, Kolmogorov-Smirnov, and Cramér-von Mises tests for further analysis, not requiring homoscedasticity assumption (Table 1). Q-Q plots comparing the distribution between EVs and parent bacterial cell counterparts for the P1 and WT samples (Fig 1. e-f) show evidence for a subset of genes less conserved in EV packaging and genes predominant in EVs. Notably, the data points, particularly near the median values, closely align with the reference line, indicating similarity in the overall distributional characteristics. However, the tails of the distribution present significant deviations from the reference line, offering evidence for a subset of genes less conserved in EV packaging and genes predominant in EVs.

To formally assess the distributional differences between the genetic components of EVs and their donors, we performed the Mann-Whitney U test to determine whether statistically significant differences exist in the median normalized gene copy counts between the experimental and simulated samples (Table 1). The p-values across all pairwise comparisons between EVs and their parent bacteria, ranging between 0.0325 and 0.9146, suggest inconclusive evidence for differences in the median distribution of gene abundances between EVs and their respective patent bacteria.

For robustness, we additionally employed the Kolmogorov-Smirnov (K-S) and Cramér-von Mises (C-V) tests to compare the empirical cumulative distribution functions (ECDFs) of the samples being compared (Fig. 1c). In all pairwise comparisons, both the K-S test and C-V test results yielded p-values significantly below the conventional α = 0.05 threshold (Table 1), indicating statistically significant evidence to reject the null hypothesis that the EVs’ and Donor’ gene counts are from the same distribution, which is analogous to evidence for nonrandom selection of donor genes packaged into EV cargos.

The two-sample Kolmogorov-Smirnov (K-S) test ^23^ calculates the maximal absolute vertical discrepancy $D_{n}$ between two empirical cumulative distribution functions (ECDFs) as:

$$D_{n}=\sqrt{n}{\;sup}_{x}\;\left|F_{n}(x)-F_{0}(x)\right|,$$

and the C-V test ^24^ employs a uniform weight function to evaluate the quadratic integrated distance $W^{2}$ between the empirical cumulative distribution functions (ECDFs) across the entire distribution spectrum, defined as:

$$W^{2}=\int {\left[F_{n}(x)-F_{0}(x)\right]}^{2}dF_{0}(x),$$

Where, n is the number of uniquely mapped genes, $F_{n}(x)$ is the ECDF from the EV sample and $F_{0}(x)$ is the ECDF from an ASF519 simulated sample (treated as the theoretical distribution).

The K-S test’s $D_{n}$ statistics indicated a maximum difference of around 0.1 normalized reads per kilobases, while the W^2^ statistics revealed roughly 30 quadratic integrated discrepancies in each pairwise comparison round. Given the difference in definition, compared to the C-V test, the K-S test is more sensitive to changes in the distribution’s center than in its tails. Hence, our result suggests evidence for more pronounced differences between the DNA copy count distribution around the distribution tails as opposed to the distribution center. QQ plots comparing the distribution between EVs and their donors’ theoretical (simulated ASF519) distributions (Fig 1. e–f) support our findings by signifying the evidence for the presence of differentially overrepresented and underrepresented subset of genes evident in the upper and lower quartiles of the normalized counts distribution.

### Identification of a robust consensus of 256 genes as being significantly differentially packaged in EVs from their parent bacteria

To ensure the reliable identification of genes that are significantly differentially packaged in EVs and from their parent bacterium, *P. goldsteinii* ASF519, we performed differential abundance (DA) analysis using two statistical models, DESeq2 ^25^ and limma-trend ^26^. DESeq2’s Negative Binomial distribution accurately models overdispersion and biological variability in our data, providing more sensitive results for low-count genes. On the other hand, limma-trend’s Gaussian distribution assumption is less impacted by over-dispersion and is more robust against false positives caused by high variability.

**Figure 2:**
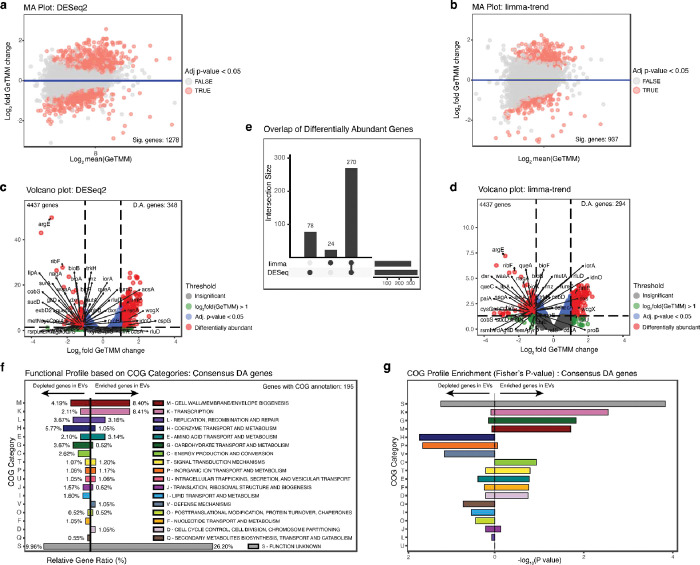
Differential abundance analysis identifies 259 differentially abundant (DA) genes robust to the choice of dispersion stabilizing and statistical model employed. The log ratio (M) vs an average (A) plot (MA plot) shows (a) 1280 genes that are differentially abundant between EVs and its donor bacterium *P. goldsteinii* ASF519 with a B-H adjusted p-value < 0.05 under the DESeq2 model and (b) 937 genes under the limma-trend model. The volcano plot segmented into threshold values of log_2_(GeTMM) fold change > 1 and B-H adjusted p-value shows (c) 350 DA genes under the DESeq method and (d) 294 DA genes under the limma-trend method, where (e) an agreement of 269 genes is shown under both regimens. (f) COG annotation among the identified DA genes under both DESeq2 and limma methods is shown in the order of the total relative gene ratio (%), where the direction of the plot signifies the sign of log_2_(GeTMM) fold change for each gene assigned to a specific category. The Fisher’s exact test-derived directional enrichment score plot (g) with gray dashed lines showing −log_10_(0.05) threshold values indicates that the identified genes assigned to the S, K, P, and M COG categories show a statistically significant enrichment or depletion in their respective COG category.

We find 1280 and 937 genes that showed differential abundance under the DESeq2 and limma-trend models, respectively, with a B-H adjusted p-value < 0.05, illustrated in the MA plot (Fig. 2a and 2b). A set of 350 and 294 genes exhibited differential abundance using a log_2_(GeTMM) fold change > 1 and a B-H adjusted p-value < 0.05 as thresholds (Fig. 2c and 2d). The consensus of 269 genes identified as significantly differentially abundant between EVs and their donor ASF bacteria (henceforth referred to as Consensus DA genes) across both DESeq2 and limma-trend models (Fig. 2e), providing strong reliability for downstream functional analysis and biological interpretations of the roles of these genes in extracellular vesicle content.

### EV cargo shows significant functional enrichment and depletion, suggesting evidence for functionally selective DNA packaging

To assess whether a distinct pattern of functional gene set enrichment or depletion exists in EVs compared to their parent bacteria, we conducted a functional gene set enrichment analysis based on the Clusters of Orthologous Groups (COG) categories. The bi-directional functional enrichment plot shows the relative magnitude and direction of functional gene enrichment and depletion in EVs vis-à-vis their parent bacteria ASF519 (Fig. 2f). Here, the gene enrichment and depletion in EVs was quantified using relative gene ratio (%) showing the proportional COG assignment amongst the identified consensus DA genes with B-H adjusted p-value of less than 0.05, where the direction of the plot signifies the sign of log_2_(GeTMM) fold change for each gene assigned to a specific category. Our findings suggest that, on average, genes in specific COG categories such as S, M, K, and T are more likely to be found in EV cargos compared to L, H, and G groups under our study’s culture and growth condition.

To validate which categories show statistically significant enrichment/depletion, we conducted a hypergeometric test (one-sided Fisher’s exact test) to calculate the enrichment score for identified genes in each COG category (Fig. 2g). We find that the identified consensus DA genes assigned to the function unknown (S), transcription (K), inorganic ion transport and metabolism (P), and cell wall/membrane/envelope biogenesis (M) categories significantly exhibit a statistically significant over/under-representation, beyond what would be expected by random probability.

**Figure 3:**
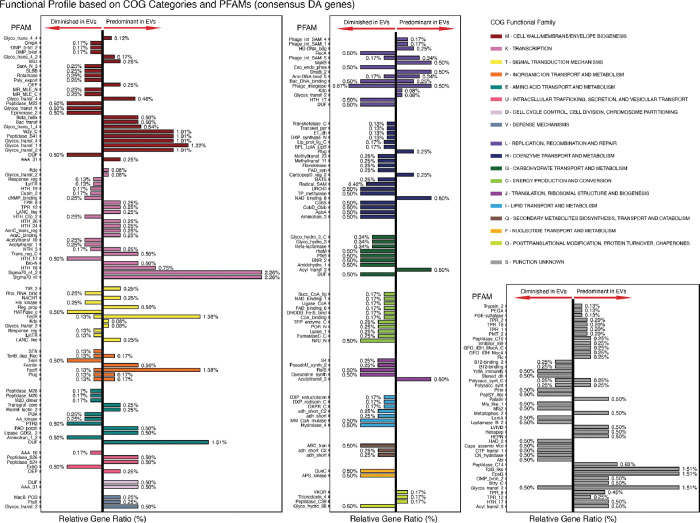
Protein Family Domain analysis, grouped by COG categories on consensus DA genes. The figure displays 259 consensus differentially abundant (DA) genes, each with a Benjamini-Hochberg (B-H) adjusted p-value of less than 0.05. These genes are further classified based on their predicted Protein Families (PFAMs) and are organized into Clusters of Orthologous Groups (COG) categories.

To gain a better understanding of the possible functional bias induced by the differential DNA packaging between extracellular vesicles and their parent bacterium, *P. goldsteinii* ASF519, we performed protein domain analysis using protein domain families (PFAMs) categorized by COGs (Fig. 3). Here, each of the consensus DA genes, each with adjusted p-value < 0.05, is mapped to its corresponding PFAMs and categorized by COGs to explore the functional characteristics and its associated protein domains. Based on the findings from the enrichment score, we focus on understanding the enrichment of genes coding for proteins in the S, K, and M COG groups and depleted genes in the P group, respectively.

The function unknown (S) category exhibits a significant enrichment in EVs relative to the parent bacterium, with a relative gene ratio of 24.04% compared to 9.13% (Fig. 2f), where both identified enrichment and depletion are shown to be statistically significant (Fig. 2g). Within this COG category of DA genes, EVs derived from ASF519 revealed a significant enrichment of genes associated with polysaccharide biosynthesis and membrane functions (Fig. 3).

We observed a noteworthy overrepresentation of EpsG, a gene implicated in exopolysaccharide polymerization ^27^, potentially associated with biofilm formation ^28^. We also find an overrepresentation of genes coding for glycosyltransferases Glycos_transf_2, Polysaccharide biosynthesis protein such as Polysacc_synt and C-terminal domain-containing Polysacc_synt_C, all implicated in polysaccharide synthesis and glycosylation processes. O-antigen ligase-related protein coding gene Wzy_C, another gene integral to polysaccharide biosynthesis, was also notably enriched in EVs. These findings propose a selective packaging mechanism in EVs for proteins involved in polysaccharide biosynthesis, hinting at the potential roles of these encapsulated genes coding for polysaccharide synthesis in EV biology and host-microbe interactions.

Additionally, we identified a significant overrepresentation of membrane-related proteins coding genes, notably OMP_b-brl_2, a gene encoding outer membrane beta-barrel proteins crucial for transport and signaling. This observation is consistent with prior research highlighting the importance of β-barrel membrane proteins in folding and integration into lipid bilayers ^27,29^. Moreover, enrichment of TolB_like proteins was noted, proteins known for their role in maintaining bacterial outer membrane integrity and potentially involved in peptidoglycan recycling or covalent linking with lipoproteins ^30^.

These findings suggest a selective mechanism in EVs for packaging genes associated with polysaccharide biosynthesis and membrane functions. This sheds new light on the potential roles of these genes in EV biology, particularly in the context of EV-microbe interactions within microbial community dynamics (Discussions).

### Overrepresentation of transcription-related protein-coding genes in EVs

The transcription (K) category exhibits a significant enrichment in EVs relative to the parent bacterium, with a relative gene ratio of 2.16% compared to 8.09% (Fig. 2f).

We identified a significant overrepresentation of Sigma 70 subunit (Sigma70_r2, Sigma70_r4_2) domains coding genes in EV cargo (Fig. 3). Sigma70_r2 is the most conserved in the sigma-70 family, crucial for −10 promoter recognition and core RNA polymerase binding ^31^. Likewise, Sigma70_r4_2 interacts with the −35 promoter element through a helix-turn-helix motif ^31,32^. Interestingly, multiple Helix-Turn-Helix (HTH) Domains (HTH_3, HTH_18, HTH_24, HTH_26, and HTH_17) are also overrepresented in EVs, pointing to a potential role in DNA binding and gene regulation. HTH_Crp_2, however, also shows an underrepresentation, indicating a selective mechanism for packaging these regulatory genes. The conspicuous enrichment of helix-turn-helix (HTH) domains in EV-enriched ORFs implicates their role in DNA binding and gene modulation ^33,34^. These domains are a staple in transcription factors, capable of specific DNA sequence interactions ^35,36^.

Sigma factors are integral elements of the RNA polymerase holoenzyme, known to guide the RNA polymerase to specific promoter sites and facilitate transcription initiation ^31,35^ Among the different classes, the sigma-70 family is particularly relevant, given its broad role in general transcription in actively expanding cells and its ability to be substituted by alternative sigma factors under specialized conditions ^37^. The enhanced prevalence of these domains in EVs suggests specialized transcriptional activity within these vesicular structures.

### Overrepresentation of cell wall/membrane biogenesis related protein-coding genes in EVs

Genes related to cell wall/membrane biogenesis (M) were found to be enriched in EVs at a relative gene ratio of 7.93%, compared to 3.85% in the parent bacterium (Fig. 2f).

Our findings reveal that EV packaging exhibits a notable prevalence of DNA copies associated with domains related to classes of Glycosyl transferases (GT) (Glycos_transf_1; Glyco_trans_1_4; Glycos_transf_2; Glyco_transf_4; Glyco_transf_4_2; Glyco_transf_4_4) (Fig. 3). GTs belonging to the membrane-associated GT-B family are known to catalyze the transfer of a sugar molecule from nucleotide-sugar donors to specific acceptor substrates that are linked to the membrane, mainly in the form of lipids and proteins ^38^. The overrepresentation of genes coding for GT domains suggests a potential role for these vesicles in modifying the surface glycans of recipient bacterial cells, which could have significant implications for cell-cell interactions and communication. We also find a significant overrepresentation of genes associated with Wzy_C domain, also overrepresented in S category ^39^, along with Wzz domain ^40^, both of which are involved in Polysaccharide biosynthesis and chain-length modification of O-antigen chains, a vital component of the lipopolysaccharide (LPS) complex in the outer membrane in Gram-negative bacteria ^41^. These findings suggest EV’s potential role in assisting the synthesis of a wide range of glycoconjugates in biological membranes, which are essential for the recognition processes between bacterial cells and the extracellular matrix.

### Preferential packaging of serine and cysteine peptidase coding genes and depletion of metallopeptidase coding genes: Protein degradation

We find that Amongst the identified DA genes, all occurrences genes coding for cysteine peptidases (Peptidase_C10, Peptidase_C39, Peptidase_C14) and Serine peptidases (Peptidase_S41, Peptidase_S26, Peptidase_S24) were shown to be preferentially packaged in EV cargo as opposed to metallopeptidases (Peptidase_M20, Peptidase_M28).

The Peptidase_S41 domain is part of the biosynthetic pathway for the secondary metabolite ustiloxin B. Given that ustiloxin B has antimitotic properties, the overrepresentation of Peptidase_S41 could indicate that EVs are specialized in the production or modification of this secondary metabolite, which could have implications for host-microbe interactions or microbial ecology ^42^. Peptidase S24 domains, notably LexA, are found to play a role in the bacterial SOS response ^43^. LexA’s self-cleavage and derepression of transcriptional activities may position EVs as specialized entities for stress mitigation. Activating stress-response pathways in recipient cells upon EV uptake corroborates this idea ^44^. Peptidase S26 domains, on the other hand, involve signal peptidases that facilitate the removal of signal peptides from proteins earmarked for the inner membrane.

The dual presence of Peptidase S24 and S26 domains in EVs likely represents a composite (integrated) regulatory system where stress response and protein secretion are intertwined. Another interesting finding is the enrichment of the Outer Envelope Protein (OEP) domain in EVs, suggesting a role in vesicular interaction and transport across the bacterial envelope (via Membrane trafficking) ^45^.

### EV-DNA content shows a locally-biased enrichment pattern associated with its genomic proximity and functionality

**Figure 4:**
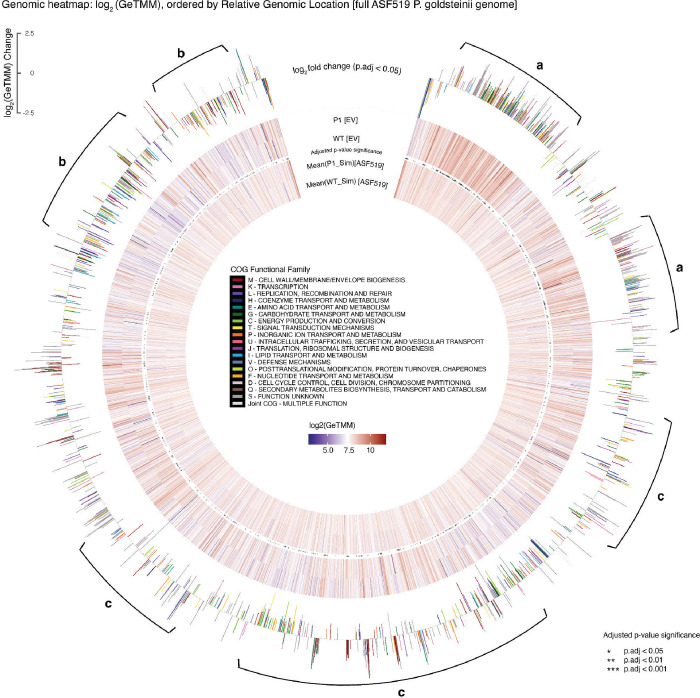
Circular genomic heatmap, ordered by relative genomic location of the gene within donor bacteria (clockwise). Outer bar plots represent the log_2_(GeTMM) fold change between −2.5 to 2.5 of genes with B-H adjusted p-value < 0.05 from DEseq2, colored by its respective COG category. (4437 filtered genes) “Joint COGs” were defined as genes mapped to multiple COG categories. We find clear (a) overrepresentation and (b) underrepresentation of genes in EVs originating from particular sections of the donor genome. When focusing on the proximity of genomic location and its function, sections of the donor genome with (c) unambiguous enrichment patterns to EV cargo show that neighboring genes with similar COG functions tended to exhibit a consistent sign of log_2_ fold change.

To investigate whether specific DNA segments of the donor genome are more likely to be encapsulated in EV-DNA cargo, we visually present the enrichment pattern and direction of DNA copy counts along the relative position on the donor genome (Fig 4). According to our analysis, DNA fragments from particular regions of the donor bacterial genome were more inclined to be contained in EV cargo (Fig. 4 a-b). Notably, focusing on the more directionally complex area (Fig. 4c), we observe that specific sets of genes located in close genomic proximity, belonging to the same COG (Clusters of Orthologous Groups of proteins) category, showed a tendency of a consistent enrichment direction. We suggest the potential for a selective DNA packaging mechanism arranged in a set of operons, neighboring genes functioning under coordinated gene regulation, for a coordinated expression of these genes.

### Preferential operon-unit EV encapsulation hypothesis

**Figure 5:**
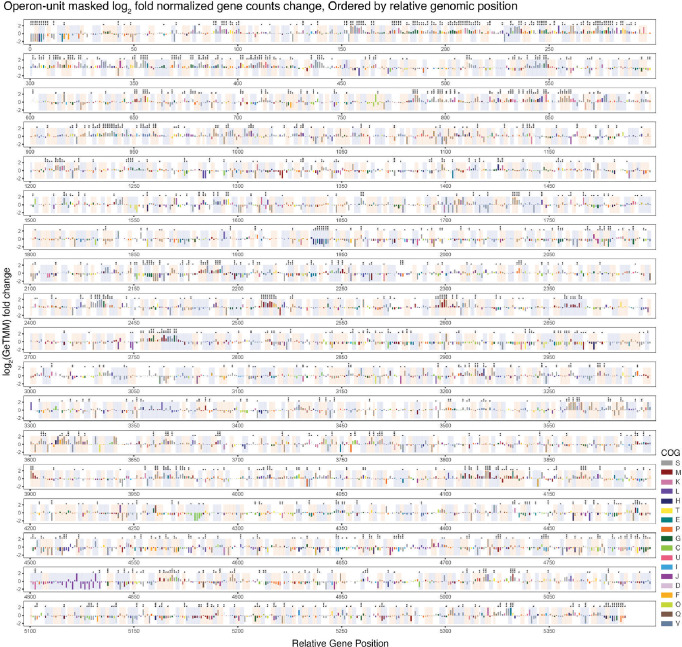
Operon arrangement of EV genes, sorted by relative genomic positions along the ASF519 genome. The figure represents each EV gene from the ASF519 genome, illustrating both the direction and magnitude of enrichment or depletion, with individual genes colored according to their assigned Clusters of Orthologous Groups (COG) categories. The operon group is masked with light amber and blue shading to distinguish the set of predicted genes within the operon structure, highlighting 1229 identified operon units, alternately overlaid with light amber and blue shading to distinguish the set of predicted genes within the operon structure. The B-H adjusted p-values from the differential abundance analysis are indicated by symbols (p < 0.001 as ***, p < 0.01 as **, and p < 0.05 as *).

To assess our hypothesis that the gene packaging mechanism of EVs is arranged in operon units, we performed operon prediction along the complete ASF519 genome sequence. We visualized the enrichment of genes along the relative genomic position log_2_ fold normalized gene count change colored by COG category, masked in alternating color between amber and blue, where each rectangular mask around groups of gene representation shows the 1229 predicted operon units within the ASF donor bacterial genome (Fig. 5). We report an observable consistency of enrichment direction within operon units, where genes, especially for those that are significantly differentially abundant and in the same COG group, exhibit the tendency to be either over-represented or under-represented together.

**Figure 6:**
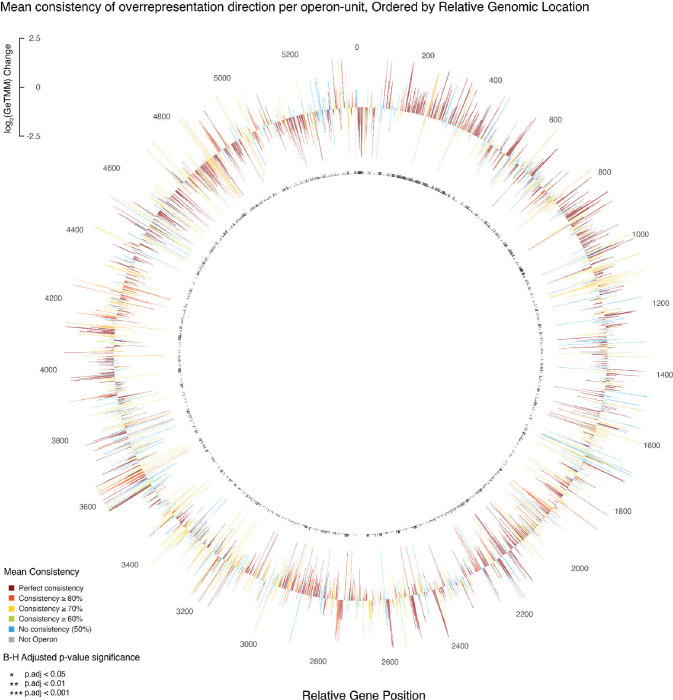
Mean consistency of overrepresentation direction of EV genes from ASF519 genome, sorted by relative genomic position. The diagram includes a representation of each gene, illustrating both the direction and magnitude of enrichment or depletion, with individual genes colored according to their assigned Clusters of Orthologous Groups (COG) categories. The operon group is masked with light amber and blue shading to distinguish the set of predicted genes within the operon structure, highlighting 1229 identified operon units, alternately overlaid with light amber and blue shading to distinguish the set of predicted genes within the operon structure. The B-H adjusted p-values from the differential abundance analysis are indicated by symbols (p < 0.001 as ***, p < 0.01 as **, and p < 0.05 as *)

To formally assess the strength of the observed pattern of gene enrichment/depletion consistency within operon units, complementary figure 5, we defined intra-operon sign-consistency as the proportion of EV-genes within an operon that display the same sign of log_2_(GeTMM) fold change. Among 1,229 operon units analyzed, 653 exhibited perfect consistency (Fig. 6). Distribution of the remaining units was as follows: 34 (80–100% consistency), 93 (70–80%), 166 (60–70%), 31 (50–60%), and 268 with 50% consistency (analogous to unobserved consistency). This finding suggests a potential for a regulated gene packaging mechanism at the operon level in extracellular vesicles.

Additionally, to test our preferential operon-unit packaging hypothesis, we assessed the statistical significance of the proposed directional consistency using a one-tailed Welch’s t-test via the nested bootstrap method. This test was designed to determine whether the mean consistency of the sign significantly surpassed 90% mean consistency. We observed a t-value of 207.75 with 999 degrees of freedom, accompanied by a p-value significantly lower than 2.2e^−16^ with the mean consistency calculated at 91.67% (Table 2). The notable mean consistency, well above the 50% baseline, indicates an evident conformity of directionality in gene abundance patterns within operons. This finding suggests a potential for regulated EV-DNA packaging mechanism at the operon level in extracellular vesicles.

**Figure 7:**
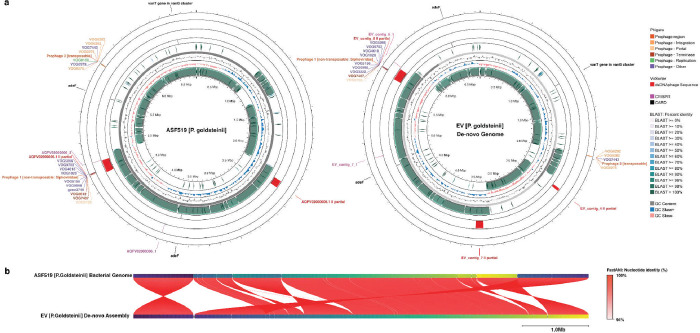
Visualization of the de-novo assembly of EV reads (left) juxtaposed against the reference donor ASF519 genome (right). (a) The Basic Local Alignment Search Tool (BLAST) results provide a comparative assessment of nucleotide sequences, highlighting their percentage identity. Regions corresponding to identified prophages, along with their predicted prophage sequences, are delineated. The FastANI analysis (b) offers a comparative genomic overview between the EVs and their respective donor genome, emphasizing the overarching scope of gene packaging from the donor bacteria to EVs.

To dissect the extent of genomic parallels between the ASF519 bacterium and its extracellular vesicles, we performed a de-novo assembly of P1-ASF519 EV sample DNA with 86.4x coverage (Supplementary Table. 4–5) for a side-by-side comparison of the DNA content between the EV cargos and the donor ASF519 genome (Fig. 7a). FASTANI analysis (Fig. 7b) revealed a nucleotide identity of 99.9772% between EV and ASF519 bacterial assemblies, with reciprocal mapping illustrated by red line segments, suggesting that EV-DNA content spans across the entire chromosome of the donor bacterium. The genomic composition of the EV cargos displayed a GC content of 43.48% and a total assembly size of 6,850,587 bp, closely mirroring the ASF519 donor bacteria’s 43.47% GC content and 6,847,904 bp size (Supplementary Table. 6). This significant genomic congruence between ASF519 and its EVs is further supported by BLAST and FASTANI results. Notably, two prophage regions were identified: a complete dsDNA prophage from the Siphoviridae family, non-transposable, was present in both assemblies, whereas in the EV assembly, only non-replication-related phage genes were detected from a transposable phage region.

## Discussion

A considerable amount of recent research has been devoted to determining and studying the role of proteins and RNAs within extracellular vesicles ^2,11,16,46–48^. In more recent years, the significance of extracellular vesicle DNA has been increasingly acknowledged in the community of microbiology ^2,46,49^, particularly in light of questioning the mechanism of DNA cargo packaging during vesiduction ^17^ and its function in genetic transfer ^13,50–52^. The results of our study indicate that the DNA copy counts in EV cargos show evidence of selective packaging in the cultured EV sample from *Parabacteroides goldsteinii* ASF519. Furthermore, we present evidence of specific genes and functional grouping of genes more prone to being included in EVs, indicating the presence of a targeted selection process in EV-DNA packaging.

Functional gene set overrepresentation analysis revealed overrepresentation of genes in the function unknown (S), transcription (K), and cell wall/membrane/envelope biogenesis (M) COG categories. A disproportionate amount of genes encoding proteins involved in exopolysaccharide polymerization, polysaccharide biosynthesis, and glycosylation processes were identified, corroborating existing research pointing to EV’s potential role in biofilm formation, microbial dynamics and host-microbe interactions ^27,28,48,51,53,54^. O-antigen ligase and membrane-related proteins potentially involved in gene transfer were also found to show relative abundance, suggesting EV’s role in signaling and maintaining receiver bacteria’s outer membrane integrity ^7,30^. Furthermore, genes coding for the Helix-Turn-Helix (HTH) and Sigma 70 subunit domains were found to be preferable constituents in the composition of EV cargos, suggesting that EVs may play a role in post-transcriptional regulation of gene expression and modulation of transcription in response to particular environmental or stress factors ^55,56^. We also note the enrichment of EV-genes coding for Peptidase S24, S26 domains and OEP domains, which may suggest that they could act as mediators of cellular adaptation, contributing to stress response and protein homeostasis within microbial communities ^57,58^. Some studies allude to the possibility of EVs serving as bioactive molecule carriers, transporting proteins and nucleic acids that can reshape cellular activities^16,48,59^. We speculate that EVs might serve as dynamic regulatory entities, possibly engaged in specialized transcriptional functions within these vesicular structures, potentially serving a direct role in interactions with microbes in its surrounding extracellular environments ^12,60–62^. Contrasting to the findings of Sartorio et al. ^48^ and Elhenawy et al.^16^ identifying EV’s specialized role as a glycan-degrader, we find a significant presence of DNA copies in EVs associated with coding for proteins in glycosyltransferase domains, particularly those from the membrane-associated GT-B family hinting at EV’s potential role in altering the surface glycans of recipient cells. However, as studies have shown the strain-specificity and the dependencies of EV cargo content on the experimental growth condition, we limit our interpretation and findings to the particular bacterial strain and experimental condition used in this study ^8,19,63^.

In concordance with previous studies on *Streptomyces coelicolor* EVs ^8^ and *Pseudomonas aeruginosa* EVs ^53^, we also demonstrate that EV-DNA cargos cover the entire genome of the bacterium. In *Pseudomonas aeruginosa* EVs, specific regions associated with virulence and antibiotic resistance were reported to be enriched ^19,53^. Our findings also support this by demonstrating evidence for a regionally enriched pattern of DNA packaging in EVs, suggesting that specific segments of the donor genome are preferentially encapsulated in EV-DNA cargo. Our research offers insights that could address the uncertainties highlighted in the two previous studies, raising questions surrounding whether the DNA components in EVs are merely fragments or complete sequences and whether there exist underlying mechanisms that explain such DNA selectivity ^8^.

The findings of our study contribute to the broader understanding of the mechanisms underlying the selective packaging of DNA in EVs. We provide novel evidence for a locally biased enrichment pattern of DNA content in extracellular vesicles, suggesting that specific segments of the donor genome are preferentially encapsulated in EV-DNA cargo. This enrichment appears to be associated with the genomic proximity and functionality of the DNA segments, indicating a potential for a strategic DNA packaging mechanism arranged in a set of operons. We demonstrate the relevance of the new perspective on the preferential operon-unit EV encapsulation hypothesis, suggesting that the gene packaging mechanism of EVs is organized in operon units. This selective DNA packaging could significantly influence the functional dynamic that is delivered to the recipient cell, affecting its behavior and potentially its genetic makeup, underlining the importance of this process in bacterial adaptation and survival.

Our operon-unit EV encapsulation hypothesis, if true, provides clues to answering many of the unanswered questions in the field. It provides evidence that EV-DNA not only has the capability of spanning the segments of the whole genome, but major genomic components of EVs are unlikely to be in the form of random DNA fragments but are more likely to be in a set of contiguous gene units. Based on the previous findings alluding to EVs’ ability to contain and transfer plasmids ^7,52,64,65^, we suggest a potential for bacterial species packaging multi-copy plasmids of containing genes in operon units in EVs, either in a circular or in an open-ended form, each hypothesizing for active gene regulation within EVs or for efficient reshaping of recipient bacterial genome through EV-mediated horizontal gene transfer (HGT). Regarding EV’s cargo for packaging plasmid content, studies have demonstrated that EVs can transfer plasmids to bacterial populations. Antibiotic stress has been noted to alter the DNA content and physical characteristics of EVs from *Escherichia coli* and *Acinetobacter baylyi* ^64^. Additionally, it has been reported that the amount of plasmid DNA per vesicle increases in mutant bacterial strains with peptidoglycan (PG) defects compared to wild types. This is particularly evident in the presence of PG synthesis inhibitors like glycine ^64,66^. Our hypothesis pointing to EV-mediated HGT mechanism based on readily uptakable operon-unit plasmid would enhance the understanding of the previous studies’ findings related to EV’s function in microbial evolution and communication with hosts and within bacterial communities through HGT ^67,68^.

It has been widely accepted that bacteriophage lysin is a significant factor in triggering an increased vesiculation and DNA quantities within EV cargos ^11,15,50,53^. Recent studies have explored the EV-DNA content concerning phage-DNA, revealing phage DNA in EV cargos, along with host and recombinant plasmids and chromosomal DNA ^7^. For example, a hyper-vesiculating mutant of *Escherichia coli* showed increased resistance to antimicrobial peptides compared to the wild-type, where the authors demonstrated EVs supplements’ interference with T4 bacteriophage infection of *Escherichia coli*, describing the potential EV’s function in defense against phage infection ^69^. Our findings corroborate these studies by identifying the complete dsDNA prophage region predicted to originate from the Siphoviridae family. Furthermore, we note that EV cargoes also contained a partial prophage region, with the exception of the replication-related gene identifiable in the donor genome. The selectivity observed could serve as a defensive mechanism employed by the host bacteria, wherein the donor exclusively packages the essential genomic segment of the prophage that is beneficial for safeguarding their bacterial community against phage infection.

Although EVs are considered reservoirs for protecting the genetic material by serving resistance against environmental stressors, questions remain whether EV-DNA composition is influenced by stressors such as DNase activity, temperature, nutrient availability, and antibiotics ^70–72^. In the context of our results, we should consider that the makeup of EV-DNA may not be strictly predetermined. Instead, specific operon gene units may be packaged in EV cargo in varying quantities in response to changes in the external environment ^73^, allowing them to carry out specific functions, thereby restricting our findings in a context-dependent manner. Additional experimental investigations are needed to validate our findings derived from our computational workflow. Additionally, we note that the genetic material sequenced for our study may not have originated exclusively from the EV cargo, as some extracellular DNA may have adhered to the exterior surfaces of the vesicles. Future research could employ DNase treatment to validate the exclusion of externally-bound DNA and distinguish EVs from cell lysis byproducts by analyzing samples taken across the growth phases of the studied culture.

## Methods

### Bacterial culture and minimal media

ASF519 and transposon-insertion mutant (insertion of “TTC GCT CTG TTC AAT A” at OIDBDKIG_02333 locus)), were cultured in the minimal medium for *Bacteroides spp*. The medium consisted of four salts and a carbon source, 13.6 g/L KH2PO4, 0.875 g/L NaCl, 1.125 g/L (NH4)2SO4, and 5.0 g/L anhydrous glucose (0.5% w/v final) in a mixture of half tap and half MilliQ filter water. The pH was adjusted to 7.2 with concentrated NaOH. The medium was further supplemented with 10 mg/ml Hermin solution ^74^ (MBD0052), 1 mL of 0.1 M MgCl2, 0.4 mg/mL FeSO4 in 10 mM HCl, 1 mg/mL vitamin K1, 0.8% w/v CaCl2 and 0.5 g/L of L-Cysteine. We modified this medium to contain an additional 10 mL of multi-vitamin (ATCC #MD-VS) and 10 mL trace mineral (ATCC #MD-TMS) supplements. The medium was filter-sterilized through a 0.22 μm filter and placed in an anaerobic chamber (Coy Labs anaerobic chambers) in a mixture of 5% CO2, 5% H2, and 90% N2 overnight for degasification. The medium was inoculated with bacteria the following day. The bacteria were grown for 72 hours or until an optical density (OD600) of approximately 0.4–1.0 was achieved. The spent medium was harvested via centrifugation at 8000 rpm at 4°C for 10 minutes and filter-sterilized with a 0.22 μm filter system.

### EV isolation

The spent culture medium was initially centrifuged to remove cellular debris and filtered sterilized by a 0.22 μm filter. Ammonium sulfate was then added at a concentration of 70% w/v to precipitate EVs, and the solution was incubated at 4°C for 12–16 hours for complete precipitation. EVs were harvested by centrifuging the medium with the precipitations at 10,000 rpm at 4°C for 30 minutes. Supernatants were decanted, and EV pellets were resuspended with PBS, then concentrated to 3ml with an Amicon Ultra-15 centrifugal filter (100 kDa MWCO)(UFC9100) unit at 3700g at 4°C for 25 minutes. The EV was filter-sterilized by a 0.22 μm syringe filter and stored at −20°C for size exclusion chromatography (SEC).

### EV purification

To obtain pure EV, after the isolation of the EV, they were purified by gel filtration. Size exclusion chromatography (SEC) was done by using the HiPrep 16/60 Sephacryl S-400 HR column and the AKTA pure 25 L1 system. The first peak (contending 20×106 Da size protein structures) was collected and concentrated with an Amicon Ultra-15 centrifugal filter (100 kDa MWCO) at 3700 rpm at 4°C for 25 minutes. The EVs were further purified by iodixanol gradient ultracentrifugation with Iodixanol solution (OptiPrep^™^ # 07820) at densities: 40% (with samples), 35%, 30%, 25%, 20%, and 10%. The gradient was centrifuged using an Optima XE-90 (Beckman Coulter) and SW41 T1 rotor at 180,000 g for 3 hours at 4°C. Factions 8–11 were collected and concentrated using an Amicon Ultra-15 (10 kDa MWCO) centrifugal filter. The concentrated samples were washed three times with 1xPBS to remove the iodixanol, and a BCA assay was used to calculate concentration.

### Genomic DNA (gDNA) isolation

Genomic DNA from isolated extracellular vesicles was extracted using the Quick-DNA Miniprep Kit, per the manufacturer’s instructions ^75^ and eluted in 200 μL Tris-EDTA (TE) buffer. DNA quantity and purity were assessed with a NanoDrop Spectrophotometer (Thermo Scientific).

### ONT rapid sequencing genomic DNA (gDNA) library preparation

Genomic DNA from EV isolates was prepared for sequencing using the SQK-RAD004 ligation sequencing kit, following Oxford Nanopore Technologies (ONT) protocol, and sequenced on FLO-MIN106 R.9.4.1 flow cells ^76^. The prepared libraries were loaded into the primed flow cell using the MinION device, following the guidelines in the ONT sequencing protocol for the SQK-RAD004 Rapid Barcoding Kit ^76^. MinION flow cells and the EXP-WSH002 Flow Cell Wash Kit were used to sequentially examine the EV samples according to the ONT’s instructions ^77^. After each sequencing run, a 60-minute incubation period was conducted using the EXP-WSH004 Flow Cell Wash Kit before transitioning to the subsequent sequencing run ^77^ (Supplementary Table 1).

### Basecalling and QC of passing reads.

Each ASF519 EV preparation was sequenced with the MinION device from Oxford Nanopore Technology (ONT). The raw signal data stored in fast5 files were basecalled using Guppy software ^78^ (v6.5.7) under the Super-accurate (SUP) model, leveraging the dna_r9.4.1_450bps_sup.cfg basecalling configuration suitable for reads generated from R9.4.1 flow cells chemistry. Basecalling was optimized using NVIDIA Tesla V100 GPUs. During this step, simultaneous initial adapter and primer trimming were performed, and the minimum Phred quality score (Q) threshold was determined based on the QC result that followed.

To assess the initial quality of the MinION sequencing runs for sufficient basecalling accuracy, we used MinIONQC ^79^ (v1.4.2). We visually examined the sequencing quality and determined the appropriate Q score threshold for the basecalling process (Extended Data Fig. 2). The mean read quality score histogram revealed two distinct clusters: one representing reads with acceptable Phred quality scores surpassing 6.5 and another with suboptimal scores around 5. To ensure the accuracy of basecalling, the minimum Phred quality score threshold in the basecalling step was set to 7. The basecalling accuracy and quality metrics of the passing reads were assessed using PycoQC ^80^ (v2.5.2) (Supplementary Table 2).

### Adapter trimming, demultiplexing and further filtering

**Extended Data Fig. 3:**
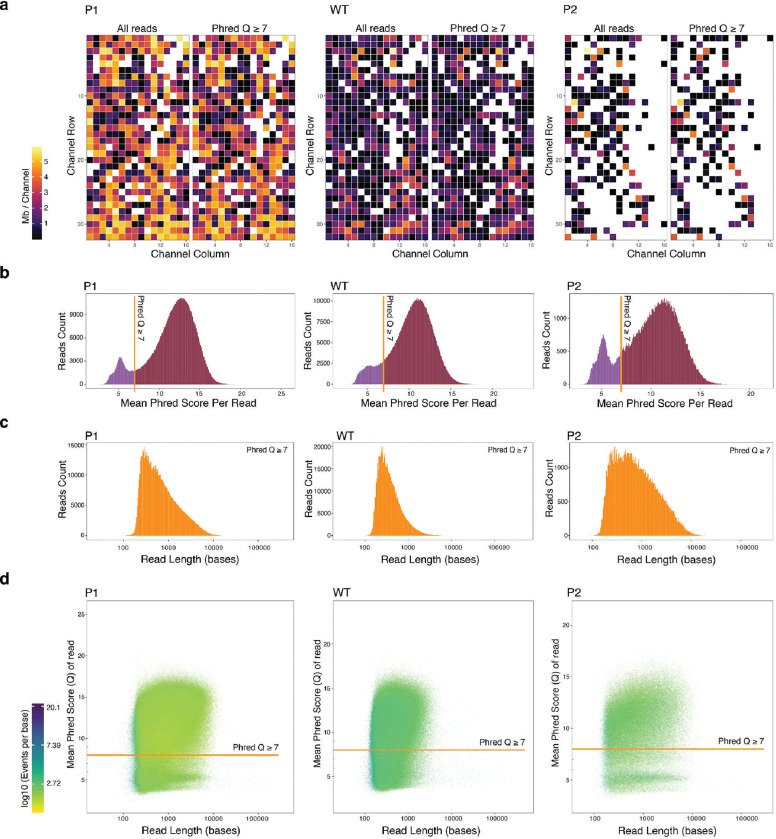
Visualized sequencing quality metrics. Customized MiniONQC script was used to visualize the sequencing qualities. (a) Visual representation of megabases sequenced per each individual channel on the R9.4.1 flow cells (512 cells per device) shows the incremental decreasing flow cell yield on subsequent sequencing rounds. (b) The histogram displays the mean Phred scores per read, aggregated from data across all flow cells. The vertical line represents the selected Q score threshold of 7, separating the reads to be filtered on the right side of the line. (c) Histograms representing the distribution of read lengths in bases on a log_10_ scale x-axis for reads passing the Phred score threshold of 7. (d) Graphical representation of the relationship between read lengths and Phred Score for each read on a log_10_ scale x-axis. Each point is assigned different colors based on the number of events per base.

Demultiplexing of Nanopore reads barcoded with SQK-RAD004 Rapid Barcoding Kit and Adapter sequence trimming of the passing reads were performed using Porechop ^80,81^ (v0.2.4). To further filter the data, reads with lengths shorter than 250 bases and reads with an average Phred score lower than 9 were removed using Nanoq ^82,83^ (v0.10.0). Final filtered reads statistics were obtained using Nanoplot ^82^ (v1.41.6) (Supplementary Table 3) (Extended Data Fig. 3).

### Re-annotation of the reference genome

To ensure accurate and complete genome annotations for further functional analysis, we utilized Prokka ^82–84^ (v 1.14.6), a tool for annotating prokaryotic genomes. The purpose of this re-annotation is not only to accurately correct any genes that may have been overlooked or mis-annotated in the original reference genome but also to ensure uniformity in analysis. Due to Prokka’s limitations in mapping functional roles and pathways and identifying operon units, we additionally incorporate complementary tools discussed in the following sections.

### Functional annotation of the reference genome

For functional annotation, we employed eggNOG-mapper ^85^ (emapper v.2.1.12–1) to align genes to the eggNOG database, deriving functional annotations and evolutionary lineage, which allows for a comprehensive functional assessment by mapping genomic loci to COG (Clusters of Orthologous Groups of proteins) and PFAMs (Protein Families and Domains). In parallel, Operon-mapper ^85,86^ was utilized to infer operon structures within the reference genome, employing an artificial neural network to evaluate intergenic distances and functional relationships of gene products, outputting ORFs, genomic coordinates, orthologous groups, and interaction networks. This combined approach ensures that our genomic analysis is comprehensive and biologically meaningful for the subsequent analyses.

### Simulated reads generation (NanoEx-Gen)

We developed a Python-facilitated pipeline NanoEx-Gen (available at: github.com/tedblry/NanoEx-Gen) specifically designed to generate simulated ONT long-reads from experimental reads, closely mimicking the error profile of the input experimental reads. This tool requires an input experimental gDNA reads file in FASTQ format alongside a reference genome file in FASTA format. The critical feature of NanoEx-Gen is its ability to introduce sequencing errors, such as mismatches, insertions, and deletions, into the sequences based on the alignments of the experimental reads to the reference genome. This approach ensures that the simulated reads generated by NanoEx-Gen accurately reflect the error characteristics inherent to the ONT Nanopore sequencing process.

NanoEx-Gen implements a three-step pipeline. The initial step, Error Profile Extraction, involves storing the length of the associated sequence in each BAM file entry, then parsing the CIGAR string for each read to determine the type and number of errors. In this context, one stands for insertions, 2 represents deletions, and four is used for soft clipping, which is treated as mismatches. The error profile is constructed as a dictionary, indicating the rate of each error type (mismatch, insertion, and deletion).

The second step, Simulated FASTQ Generation, coordinates the entire simulation process. After loading the reference genome in FASTA format, the pipeline selects random starting positions within the reference genome for each sequence in the input FASTQ file. If the chosen start position and read length extend beyond the genome’s end, the sequence wraps around to the beginning, accommodating the assumption of a circular genome. During the Error Introduction sub-step, a random number between ranges [0.0, 1.0) is generated for each base in the sequence. If this number is less than the mismatch rate from the error profile, a mismatch is introduced by replacing the base with another random base. If the number falls within the range of the mismatch rate and the mismatch rate plus the insertion rate, an insertion is introduced. If the number lies within the range of the mismatch rate, insertion rate, and deletion rate, the base is deleted. This method ensures that the probability of introducing each type of error is proportional to its rate in the error profile extracted from the BAM file. Based on the returned sequence with introduced errors, the Simulated FASTQ Generation step produces a simulated sequence by writing to the output FASTQ file along with the original sequence name and quality score. For each randomization step, a random seed is set for reproducibility.

For this study, NanoEx-Gen simulation of the reference genome enables a robust statistical comparison of read counts between experimental reads derived from the EVs produced by ASF519 sequenced using Nanopore and the ASF519 reference genome sequenced using the whole-genome shotgun sequencing method via Illumina technology. This approach also ensures that the reads generated by NanoEx-Gen are a robust representation of the anticipated output if the reference genome sequence, originally sequenced via a distinct method, were to be sequenced using ONT Nanopore. Consequently, it creates an environment conducive to undertaking comparative analyses between genomes derived from diverse sequencing platforms, reflecting the experimental conditions inherent to the ONT Nanopore sequencing process. Three sets of simulated reads were generated for each experimental sample using the NanoEx-Gen tool. The input *Parabacteroides goldsteinii* ASF519 reference genome (Version AQFV02000006.1) was retrieved from NCBI RefSeq with accession id GCF_000364265.2 ^87^. Reproducibility across the simulations was ensured by establishing a set seed value — specifically, 519, 5190, and 51900 — for each simulation run.

### Read alignment and counting

We carried out mapping and counting of simulated and EV reads using our Python-based pipeline MappCountFlow (available at: github.com/tedblry/MappCountFlow). Initially, sequencing reads were aligned to the reference genome using Minimap2 ^88^ (v2.26-r1175) aligner with adjustments for long-read specific error rates, as per Oxford Nanopore Technologies (ONT) presets (-x map-ont). The alignment utilized 30 threads, producing SAM files. Subsequently, the pipeline incorporates SAMtools^89^ (v1.18), converting these files to indexed Binary Alignment/Map (BAM) format, sorting and indexing them by chromosomal coordinates. Read counting was conducted via HTSeq-count ^90^ (v2.0), considering read name and chromosomal position. For HTSeq compatibility, gene annotations in GTF format were sorted in line with chromosome and position using Bedtools ^91^ (v2.31.0). The pipeline features the MappingStatisticsExtractor.py module that compiles a summary of key alignment metrics, such as total sequence length, counts of mapped and unmapped reads, duplication rates, and average sequence quality, providing a detailed overview of the mapping and counting results (Supplementary table 4).

### Normalization of mapped read count

For inter-sample gene-level statistical analysis, we identified that commonly used normalization methods like TMM in edgeR ^92^ and RLE in DESeq ^25,93^ do not incorporate gene length correction, leading to potential inaccuracies in intra-sample analysis. While TPM, FPKM, and RPKM attempt to address this, they also carry inherent biases ^94^, highlighting the need for a more comprehensive approach.

Our normalization strategy, addressing library size, sequencing depth, and gene length variations, utilized the GeTMM approach ^94^. This method combines gene-length correction from RPKM with the TMM normalization factor from edgeR ^94^. The integration of the GeTMM normalization strategy commenced with the computation of Reads Per Kilobase (RPK) for each gene, followed by an adjustment of the library size via TMM normalization factors, employing the formula: GeTMM = RPK * TMM Factor, where RPK = Number of reads / Gene length in KB.

### Pre-filtering and exclusion criteria for differential abundance analysis and functional characterization

To ensure accurate results in copy number differential abundance analysis and avoid false positives caused by low-coverage associated variance, we excluded 950 genes. This was done by removing four genes with incomplete annotation and genes that were present in less than two-thirds of the samples with a count of less than 100 RPK. The post-filter data represents 4437 genes that consistently appear in the dataset, indicating their reliable presence rather than sequencing artifacts. Subsequent to the RPK calculation, the data was normalized using the TMM method, adjusting for compositional differences between samples to generate the previously mentioned GeTMM normalized counts. We follow the identical data preparation pipeline for operon consistency analysis on the full 5387 mapped genes, excluding the 4 genes with incomplete annotation.

### Overall distributional comparison (pairwise test between experimental vs simulated reads)

To compare variances between experimental (EV-derived) and simulated (donor bacteria-derived) groups in P1 and WT categories, we utilized Bartlett’s ^95^ and Levene’s tests ^89^, the latter guiding our choice of subsequent tests due to its effectiveness with non-normal distributions. Based on Levene’s test outcomes, we adopted non-parametric methods such as Mann-Whitney U, Kruskal-Wallis, Kolmogorov-Smirnov, and Cramér-von Mises for distributional analysis. We organized data into vector pairs representing various genomic conditions and simulations. The Mann-Whitney U test evaluated differences between unpaired groups, while the Kruskal-Wallis test compared three or more independent samples. The Kolmogorov-Smirnov test, with a set seed of 519 to ensure reproducibility, assessed the similarity between distributions, and the Cramér-von Mises test evaluated the goodness of fit.

### Differential abundance analysis methods

To identify differentially abundant genes in EVs from their donor bacterium, *P. goldsteinii* ASF519 genome, we compared EV and donor bacteria groups using DESeq2 ^25^ and limma-trend ^26^ on GeTMM normalized counts. In the DESeq2 pipeline, a negative binomial distribution model was fitted to rounded GeTMM for its suitability in overdispersed data, and a “local” model was employed for dispersion estimation. It modeled gene abundance with a Negative Binomial distribution, accounting for biological variability. DESeq2 internally managed variance stabilization, and Wald’s test was used for hypothesis testing. Limma-trend, initially for microarray data, uses linear models with empirical Bayes moderation for log-fold change estimation, ideal for datasets with limited replicates. In the Limma pipeline, a linear model was fitted to compare EV groups with their simulated donor bacterial counterparts, using log_2_(GeTMM) values. Empirical Bayes statistics were employed to stabilize variance estimates across genes. Genes were deemed significantly differentially abundant if they exhibited an adjusted p-value (Benjamini-Hochberg method) below 0.05 and a log_2_ fold change (GeTMM) above 1. In cases where DESeq2 and limma-trend consistently identified overlapping genes as significantly over/underrepresented, we defined significant differential abundance as genes identified by both methods.

### Functional profiling (Protein Family and Domain) and COG classification

Using the eggNOG gene annotation database, we annotated each gene to ensure that the resultant differentially abundant genes could be contextualized within their broader functional landscape. The annotation data included locus tags, gene names, COG (Clusters of Orthologous Groups of proteins) categories, and PFAMs (Protein Families and Domains). The COG categories provided insights into the functional distribution of the dataset. The PFAM information facilitated protein domain analysis and provided concise functional descriptions for each gene. To handle cases where one gene is associated with multiple COG categories or PFAM domains, we adopt a relative gene abundance approach to distribute the GeTMM count across the multiple categories or domains. For cases of one-to-many mapping of genes to COGs or PFAMs, we assigned each mapped category or domain with an equal fraction of the original GeTMM counts, then computed relative abundance by dividing the relative GeTMM count by the number of classifications associated with the gene. Quantifying the proportion of genes in a particular category relative to the total number of genes allowed us to conserve the distribution of the gene function for each of the samples in comparison accordingly.

Hence, we define and compute the relative gene ratio for a specific gene in a particular domain as the following:

$$Relative\;Gene\;Ratio=(\frac{\mathrm{Sum}\;\mathrm{of}\;\mathrm{relative}\;\mathrm{GeTMM}\;\mathrm{counts}\;\mathrm{mapped}\;\mathrm{to}\;\mathrm{particular}\;\mathrm{domain})}{\mathrm{Total}\;\mathrm{GeTMM}\;\mathrm{counts}\;\mathrm{mapped}\;\mathrm{to}\;\mathrm{domains}}\}\cdot 100$$

We calculated the relative gene ratio for each significantly differentially abundant gene to identify the functional profile difference and its magnitude between the EVs and their donor bacteria.

### COG functional analysis: Fisher’s exact test and enrichment analysis

Fisher’s exact test was employed to assess gene distribution across COG functional categories between EVs and their parent bacterium. This involved constructing 2×2 contingency tables for each COG category using a custom R function. The tables included counts of specific genes in EVs, total genes in EVs, total genes in the parent bacterium, and total genes not in any category. We executed Fisher’s Exact Test for each contingency table using R’s fisher.test function. Subsequently, we transformed these p-values into enrichment scores using the negative logarithm base 10. This transformation accentuates the impact of low p-values, enabling easier differentiation between varying levels of significance. In this context, a higher −log_10_(p-value) score indicates more significant evidence against the null hypothesis, suggesting that the observed distribution of genes in the COG category is not by random chance. Conversely, a lower score implies weaker evidence against the null hypothesis, indicating that the distribution could likely be due to random variation.

### Operon mean consistency analysis

We conduct our operon-based analysis on the 1229 predicted operon units across the whole genome of the ASF519 that maps to the 5391 pre-filtered gene set. We utilized nested bootstrap resampling to assess the sign consistency of log_2_ fold change across the operon units that were identified. Our methodology consisted of two bootstrap sampling layers. We omitted operon units that contained no more than two genes. We conducted 1,000 primary bootstrap sampling iterations resampled with replacement, preserving the inherent structure of the operons conducted 1,000 primary bootstrap sampling iterations. A secondary bootstrap sampling procedure was performed within each primary bootstrap sample, resulting in the generation of 100 inner bootstrap samples. The sign consistency of the EV-gene enrichment was computed for every inner bootstrap sample. We define sign-consistency as the proportion of EV-genes within an operon that displays the same sign of log_2_(GeTMM) fold change. This calculation was performed for each secondary bootstrap sample, producing a distribution of consistency scores. The significance of the observed gene expression consistency was determined using a one-tailed t-test. The null hypothesis was examined to determine whether the mean consistency score exceeded 0.90, which would indicate a directionally consistent and highly non-random pattern in gene expression among the operon units. For each sampling round, a seed of 519 was set for reproducibility.

### DeNanoPolish: comprehensive long-read de-novo assembly pipeline

We developed DeNanoPolish (github.com/tedblry/DeNanoPolish), a Python-based pipeline that streamlines genome assembly from long-read sequencing data. Flye, Minimap2 ^96^ (v2.26, r1175), Racon ^97^ (v1.5.0), Medaka (v1.10.0) (github.com/nanoporetech/medaka), and QUAST ^98^ (v5.2.0) collaboratively function for assembly, error correction, consensus polishing, and quality assessment.

The assembly process commences with Flye constructing an initial genome assembly from FASTQ files. This is followed by iterative error correction using Racon, where original reads are aligned to the assembly with Minimap2 and refined using Racon. Each round aims to refine the assembly until no further quality improvements are observed. Medaka is then employed for final consensus sequence polishing, enhancing the assembly quality. Each iteration of assembly and polishing is evaluated using QUAST, focusing on metrics such as N50 and misassembly count. Should the assembly quality decrease post-polishing, the implemented rollback mechanism reverts the assembly to its previous state, ensuring the highest quality output at each stage.

From the polished genome and accompanying quality metrics (Supplementary Table 5), we conducted a manual examination to determine the presence of the specific insertion sequence “TTC GCT CTG TTC AAT A” within the transposon-insertion mutant EV assembly. Our analysis revealed that this insertion sequence was retained up to the fifth round of polishing. Consequently, the final consensus genome was established by implementing Medaka post the fifth iteration of Racon (Supplementary Table 6).

### DenovoContigSort: ordering and renaming contigs based on a reference genome

The DenovoContigSort (github.com/tedblry/DenovoContigSort) python pipeline was developed to facilitate the organization of contigs in de-novo assemblies relative to a reference genome. The pipeline initiates with NCBI BLASTN ^99^ search, aligning contigs against the reference genome and identifying homologous sequences. The BLASTN output is sorted based on the query sequence’s start position. Contig IDs are extracted from this sorted output and written to a file. The seqtk ^100^ (v.1.4-r122) tool subsequently reorganizes the contigs according to the order of IDs and creates an intermediate file used for the final reordering. The script generates two files: one maintaining the original contig names and another with contigs renamed using a specified prefix or the default “contig” prefix.

### Assembly visualization

We used Proksee ^101^ to visualize the genome map of pre-assembled contigs from EVs and the reference genome. The assembly was annotated using BLAST+ ^99^ (v.2.12.0) for sequence comparison, Phigaro ^102^ (v.2.3.0) for prophage regions, CRISPRCasFinder (v.4.2.20) for Clustered Regularly Interspaced Short Palindromic Repeats (CRISPR) and CRISPR-associated nucleases (Cas), Resistance Gene Identifier (RGI)(v.6.0.2) for predicted antibiotic resistome from the Comprehensive Antibiotic Resistance Database (CARD) ^103^, VirSorter2 ^104^ (v.2.2.4) for dsDNA phage sequence. Average Nucleotide Identity and alignment between EV and donor Bacteria assembly were compared using FastANI ^105^ (v.1.3.3).

## Extended Data

**Extended Data Fig. 4:**
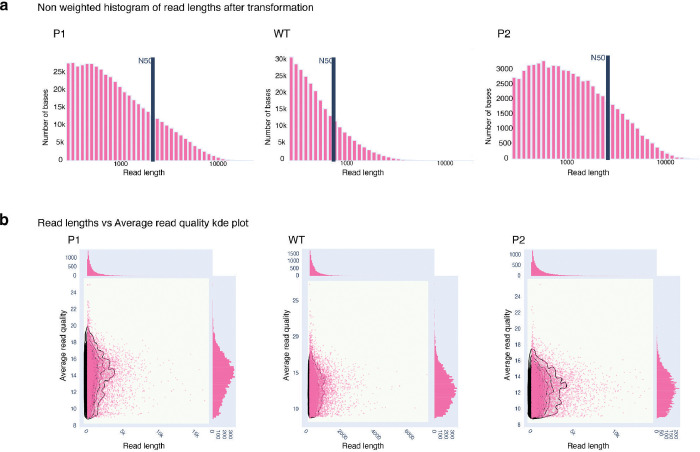
Additional sequencing QC plots. (a) Histogram showing the distribution of QC-passed read length in bases for each of the samples post log transformation with N50 metrics. (b) The kernel Density Estimation plot shows the distribution of the mean Q score and read length for each of the sample sequences.

## Supplementary Material

Supplement 1

## Figures and Tables

**Table 1:** Distributional comparison statistics for two-sided non-parametric tests (p-values rounded to 4 decimal points)

Samples (Pairwise)	Mann Whitney (U) statistic	p-value	Kolmogorov-Smirnov (D_n_) statistic	p-value	Cramér-von Mises (W^2^ statistic)	p-value
p1 vs p1_sim1	9592384	0.0374	0.097137705656975	2E-04	30.995371100547	1E-04
p1 vs p1_sim2	9585526	0.0325	0.095560063105701	2E-04	32.201184804017	1E-04
p1 vs p1_sim3	9615091	0.0584	0.087897227856659	2E-04	27.220468828898	1E-04
wt vs wt_sim1	9799139	0.7132	0.111336488618435	2E-04	37.840360189523	1E-04
wt vs wt_sim2	9828775	0.9030	0.109082713545187	2E-04	36.388224820183	1E-04
wt vs wt_sim3	9830541	0.9146	0.110660356096461	2E-04	34.463133253268	1E-04

**Table 2:** Welch t-test result for mean within-operon sign-consistency.

Statistic	Value
alternative hypothesis	μ > 0.9
t-statistics	204.74
Degrees ofFreedom	999
p-Value	< 2.2E-16
95% Confidence Interval	0.9165461 to Inf
Mean Consistency	0.9166802

## Data Availability

The Nanopore sequencing data, simulated reads, draft genome assembly has been made available in the figshare repository, accessible via Figshare (DOI: 10.6084/m9.figshare.24665244). The *Parabacteroides goldsteinii* ASF519 reference genome utilized in this study was sourced from the NCBI RefSeq database, with the accession number GCF_000364265.2, and can be accessed at [NCBI RefSeq](ncbi.nlm.nih.gov/nuccore/AQFV02000006.1).
