# Supplementary material for: DNA characterization reveals potential operon-unit packaging of extracellular vesicle cargo from a gut bacterial symbiont
Source: Res Sq. 2023 Dec 4:rs.3.rs-3689023. Preprint. [Version 1] doi: 10.21203/rs.3.rs-3689023/v1 (PMC10723553; doi:10.21203/rs.3.rs-3689023/v1)
Supplement: Supplement 1 [file NIHPPrs3689023v1-supplement-1.pdf]

Supplementary Information

Samples	Estimate ng/uL	DNA ng/µL	DNA A260/A280	Total ng
WT	236.5	6.5	1.1	45.5
P1	140.5	134.1	1.66	1072.8
P2	22.3	13.5	1.46	87.75

Supplementary Table 1: gDNA concentrations

Experiments	Reads	Bases	Median Read Length	N50 Length	Median Read Quality	Active Channels	Run Duration

P1	852245	865541374	548	1735	12.02	438	6.61
P1 (passed)	764506	780866151	558	1758	12.35	429	6.61
WT	787753	378092582	324	556	10.58	431	25.44
WT (passed)	693120	322544731	326	538	10.92	413	25.44
P2	131470	159792212	646	2248	10.38	182	27.96
P2 (passed)	108968	133540866	664	2241	10.97	157	27.95

Supplementary Table 2: Summary sequencing quality metrics

	P1	WT	P2 (for benchmarking pipeline)
Megabases of reads above QC cutoffs			
>Q7	508616 (100.0%) 620.5Mb	270931 (100.0%) 164.6Mb	68171 (100.0%) 100.4Mb
>Q10	483008 (95.0%) 589.2Mb	244654 (90.3%) 148.5Mb	60100 (88.2%) 88.8Mb
>Q12	398274 (78.3%) 489.0Mb	167218 (61.7%) 101.8Mb	38593 (56.6%) 57.2Mb
Summary Statistics			
Mean read length	1,219.90	607.6	1,472.20
Mean read quality	12.8	11.8	11.5
Median read length	706	440	887
Median read quality	14	12.7	12.4
Read length N50	2,010	693	2,440
Total bases	620,484,241	164,611,352	100,361,858

Supplementary Table 3: Filtered reads statistics and assessment

	P1	WT	P2	P1_sim1	P1_sim2	P1_sim3	WT_sim1	WT_sim2	WT_sim3	P2_sim1	P2_sim2	P2_sim3
Seed value	N/A	N/A	N/A	519	5190	51900	519	5190	51900	519	5190	51900
Type	DNA	DNA	DNA	DNA	DNA	DNA	DNA	DNA	DNA	DNA	DNA	DNA
raw total sequences	508616	270931	68171	508616	508615	508616	270931	270929	270931	68171	68171	68171
sequences	508616	270931	68171	508616	508615	508616	270931	270929	270931	68171	68171	68171
1st fragments	508616	270931	68171	508616	508615	508616	270931	270929	270931	68171	68171	68171
reads mapped	472663	143804	67273	506511	506429	506442	269336	269306	269372	67892	67894	67895
reads unmapped	35953	127127	898	2105	2186	2174	1595	1623	1559	279	277	276
reads duplicated	0	0	0	0	0	0	0	0	0	0	0	0
reads MQ0	4044	1682	532	5226	5268	5273	3464	3529	3570	601	648	662
reads QC failed	0	0	0	0	0	0	0	0	0	0	0	0
non-primary alignments	21232	8353	3094	26940	27402	27546	16752	17025	17320	3328	3515	3540
supplementary alignments	2374	351	307	510	552	578	80	78	74	107	98	70
total length	620484241	164611352	100361858	614210749	614203855	614208689	160830743	160830879	160833516	99245176	99246473	99242822
total first fragment length	620484241	164611352	100361858	614210749	614203855	614208689	160830743	160830879	160833516	99245176	99246473	99242822
bases mapped	597647501	83038831	99093375	612520521	612460182	612456146	160037073	160046289	160060580	98993694	98982804	98986650
bases mapped (cigar)	591890760	81435684	98056233	610666522	610553513	610640703	159533005	159576499	159575323	98641991	98651450	98655309
mismatches	28704754	5929708	6725548	25837331	25835234	25829429	10963922	10957150	10964709	5407114	5401417	5401542
error rate	4.85E-02	7.28E-02	6.86E-02	4.23E-02	4.23E-02	4.23E-02	6.87E-02	6.87E-02	6.87E-02	5.48E-02	5.48E-02	5.48E-02
average length	1220	608	1472	1208	1208	1208	594	594	594	1456	1456	1456
average first	1220	608	1472	1208	1208	1208	594	594	594	1456	1456	1456

	P1	WT	P2	P1_sim1	P1_sim2	P1_sim3	WT_sim1	WT_sim2	WT_sim3	P2_sim1	P2_sim2	P2_sim3
fragment length												
maximum length	19842	19190	18831	19650	19633	19642	18745	18738	18788	18627	18591	18603
maximum first fragment length	19842	19190	18831	19650	19633	19642	18745	18738	18788	18627	18591	18603
average quality	21.9	20.6	19.7	21.9	21.9	21.9	20.6	20.6	20.6	19.7	19.7	19.7
Coverage	86.43386	11.89206	14.31916	89.17568	89.15918	89.17191	23.29662	23.30297	23.30280	14.40470	14.40608	14.40664

Supplementary Table 4: Read mapping statistics from SAMtools stats result (post re-annotation, via MappCountFlow)

Rounds	Racon_0	Racon_1	Racon_2	Racon_3	Racon_4	Racon_5	Racon_6	Racon_7	Racon_8	Racon_9
N50	1994616	1993810	1993171	1992030	1991882	1990306	1988827	1988287	1987467	1985983
L50	2	2	2	2	2	2	2	2	2	2
# misassemblies										
# contigs (>= 0 bp)	14	14	14	14	14	12	12	12	12	12
# contigs (>= 1 kbp)	14	14	13	10	10	10	10	10	10	10
# contigs (>= 5 kbp)	10	10	9	9	9	9	9	9	9	9
# contigs (>= 10 kbp)	9	9	9	9	9	9	9	9	9	9
# contigs (>= 25 kbp)	8	8	8	8	8	8	8	8	8	8
# contigs (>= 50 kbp)	8	8	8	8	8	8	8	8	8	8
Total length (>= 0 bp)	6874631	6868953	6865736	6859623	6856567	6851056	6844170	6841086	6835157	6829461
Total length (>= 1 kbp)	6874631	6868953	6864939	6856729	6853940	6850061	6843191	6840111	6834216	6828515
Total length (>= 5 kbp)	6868389	6864174	6856697	6852018	6849398	6845499	6838621	6835581	6830126	6824423
Total length (>= 10000 bp)	6862869	6858538	6856697	6852018	6849398	6845499	6838621	6835581	6830126	6824423
Total length (>= 25000 bp)	6851834	6847736	6846045	6841524	6838624	6834933	6828212	6825268	6820038	6814196
Total length (>= 50000 bp)	6851834	6847736	6846045	6841524	6838624	6834933	6828212	6825268	6820038	6814196
# contigs	14	14	14	14	14	11	11	11	11	11
Largest contig	2.26E+06	2.26E+06	2.26E+06	2.26E+06	2.26E+06	2.26E+06	2.25E+06	2.25E+06	2.25E+06	2.25E+06
Total length	6874631	6868953	6865736	6859623	6856567	6850587	6843718	6840631	6834735	6829035
GC (%)	43.46	43.46	43.46	43.46	43.47	43.48	43.48	43.48	43.49	43.49
N90	343865	343688	343726	343592	343145	342757	342326	341984	341424	341090
auN	1514335.5	1514119.5	1514513.2	1513685.8	1513444	1512991.4	1511833.1	1511401	1511060.8	1509909.1
L90	6	6	6	6	6	6	6	6	6	6
# N's per 100 kbp	0	0	0	0	0	0	0	0	0	0

Supplementary Table 5: Assembly and polishing result

**Assembly**	**polished_assembly (ASF519-EV)**	
**# contigs (>= 0 bp)**	12.0	1
**# contigs (>= 1000 bp)**	10.0	1
**# contigs (>= 5000 bp)**	9.0	1
**# contigs (>= 10000 bp)**	9.0	1
**# contigs (>= 25000 bp)**	8.0	1
**# contigs (>= 50000 bp)**	8.0	1
**Total length (>= 0 bp)**	6851056.0	6847904
**Total length (>= 1000 bp)**	6850061.0	6847904
**Total length (>= 5000 bp)**	6845499.0	6847904
**Total length (>= 10000 bp)**	6845499.0	6847904
**Total length (>= 25000 bp)**	6834933.0	6847904

**Total length (>= 50000 bp)**	6834933.0	6847904
**# contigs**	11.0	1
**Largest contig**	2256246.0	6847904
**Total length**	6850587.0	6847904
**GC (%)**	43.48	43.47
**N50**	1990306.0	6847904
**N90**	342757.0	6847904
**auN**	1512991.4	6847904
**L50**	2.0	1
**L90**	6.0	1
**# N's per 100 kbp**	0.0	0

Supplementary Table 6: Final assembly result (via QUAST):

Supplementary Notes

Differential abundance analysis methods

Our analytical strategy was designed to address the unique characteristics of our Nanopore gDNA-derived gene copy count data. The aim was to identify regions and functions of the differentially abundant genes between the experimental group (P1, WT) derived from EVs and the simulated group from host bacteria. We began our analysis by comparing the results from limma-trend and DESeq2 on GeTMM normalized counts.

Two statistical frameworks were employed to robustly identify differentially abundant genes between extracellular vesicles and their donor bacterium, *P. goldsteinii* ASF519: DESeq2 and limma-trend.

DESeq2 uses a negative binomial distribution to model the count data, particularly for overdispersed data. The method employs a maximum likelihood approach for dispersion estimation. Notably, in our implementation, we utilized the "local" model fitting type for estimating dispersions, which is designed to be more robust for smaller datasets and allows for improved control of the type I error rate.

In our context, DESeq2 models the GeTMM normalized gene abundance level Y_{ij} for the gene i in a sample j using a Negative Binomial distribution:

$$Y_{ij} \sim NegBin(\mu_{ij}, \alpha_i)$$

where $\mu_{ij} = s_i q_{ij}$ represents the expected GeTMM normalized gene count, where s_j is the size factor for a sample j and q_{ij} is the normalized mean expression of gene i, and α_i is the dispersion parameter for gene i, which accounts for biological variability. In the context of gene abundance, μ_{ij} signifies the expected gene count for a particular gene i in the sample j and α_i encapsulates the inherent variability across biological replicates for gene i .

Contrastingly, limma-trend is built on linear models and was developed for microarray data. The method employs empirical Bayes to moderate the standard errors of the estimated log-fold changes. This becomes particularly useful when the dataset contains a limited number of replicates.

limma-trend models the gene abundance level Y_{ij} for the gene i in the sample j is modeled linearly to estimate the variability as:

$$y_{ij} = \beta_{0i} + \beta_{1i} x_{1j} + \dots + \beta_{pi} x_{pi} + \epsilon_{ij}$$

Here, β_{ki} are the coefficients that measure the effect of a predictor variable x_{kj} on gene i , and ϵ_{ij} is the error term representing unexplained variability.

In terms of gene abundance, the β coefficients indicate the extent to which the gene abundance level changes in response to the predictor variables, which are the experimental conditions, specifically EV reads and Host bacterial reads. The error term ϵ_{ij} represents the unexplained variability. Genes displaying adjusted p-value (via the Benjamini-Hochberg method) less than 0.05 and a $\log_2$(GeTMM) fold change greater than one were considered significantly differentially abundant. Given that a consistent pattern emerged between the two methods and a significant number of overlapping identified significantly over/underrepresented genes, we decided to further evaluate genes identified as significantly differentially abundant from both methods.

Assessment of Justification to study's choice of normalization methods and simulator/pipeline by introducing P2 sample (poor-sequencing quality sample)

For an unbiased statistical comparison of gene count distribution, incorporating a normalization regimen for read counts is crucial. In assessing our normalization method and the validity of our control-group generating simulator NanoEx-Gen, we deliberately include poor sequencing data, which we refer to as P2. A robust normalization factor computation is highlighted by a linear relationship between the sequencing depth factor and the total count for each sample, allowing GeTMM normalization-based differential abundance analysis to be both precise and accurate (Extended Data Fig 2a).

An exploratory investigation on 5391 unfiltered mapped reads was conducted alongside a comprehensive statistical assessment on 4437 filtered reads. Controlled for variations attributable to group effects, sequencing depth factors, as determined by edgeR, were plotted against the total read counts for each sample (Extended Data Fig. 2a). Within-group total counts showed minimal divergence between simulated and empirical samples, with a notable exception in the WT cohort. This divergence in the WT sample is attributable to the shorter mean read length observed during the ONT miniON sequencing step (Supplementary Table 3). For an unbiased statistical comparison of gene count distribution, incorporating a normalization regimen for read counts is crucial. A robust normalization factor computation is highlighted by a linear relationship between the sequencing depth factor and total count for each sample, allowing GeTMM normalization-based differential abundance analysis both precise and accurate (Extended Data Fig. 2a).

The $\log_2$(GeTMM) normalized gene copy counts are represented in a heatmap, revealing consistent hierarchical clustering of samples within each group (P1, WT) (Extended Data Fig. 2b). While the P1 and WT groups manifest predominantly similar gene abundance patterns for simulated samples, the P2 group displays distinct variability even within its simulated samples. A direct correlation emerges between variability in read counts and sequencing depth, with limited sequencing depth causing increased variability in mapped read counts. Such limitations often lead to shorter reads being unmapped, resulting in some genes having zero count values, more likely due to sequencing limitations than biological significance. This is particularly evident when comparing genes from P1 and P2 samples, both originating from gDNA preparations of P1-ASF519 EV biological replicates (Extended Data Fig. 2b). Principal component analysis further confirms that both experimental and simulated samples from P1 and WT cluster together, contrasting with P2 samples which scatter along the 2nd principal component axis (Extended Data Fig. 2c).

The GeTMM normalized counts distribution, visualized through violin plots, illustrates experimental groups' pronounced variance in normalized counts when juxtaposed against their simulated counterparts. The P2 reads exhibit a slightly downward skew in their central tendencies compared to P1 and WT reads, a consequence of the elevated zero count due to limited sequencing depth. The violin plot's breadth further emphasizes that simulated reads display a denser distribution near their mean values (Extended Data Fig. 2d).

Density plots of the $\log_2$(GeTMM) normalized values reveal a striking resemblance within biological groups, characterized by closely aligned patterns and distances, attesting to the Nano-ExGen simulator's efficiency, and hinting at a congruent gene copy count between the EVs and their parent bacteria (Extended Data Fig. 2e).

From a biological standpoint, parallels in gene copy counts between EVs and their parent bacteria may indicate significant genetic material retention during EV formation, potentially signifying a faithful encapsulation of genetic information.

Sequencing and basecalling quality assessment reveal varying reliability of sequenced samples in different analytical contexts.

A detailed visual inspection of the quality of MiniON sequencing runs was conducted via customized MinIONQC for sequentially arranged P1 WT and P2 EV samples (Extended Data Fig. 3). A diminishing yield over successive sequencing runs was observed, starting with the initial library load (P1 run), followed by a reloaded post-wash load (WT run), and the final load after another wash (P2 run). The diminishing yield through consecutive sequencing cycles can be observed. Histograms of mean Phred quality scores highlight a set of basecalled reads with Phred scores above 6.5, centered around a score of 13 and another subset around a score of 5 (Extended Data Fig. 3b). The Phred quality score (Q) is logarithmically determined by the probability of base-calling errors (P) as Q =

$-10 \cdot \log_{10}(P)$. A Phred threshold of 7 was established for accurate DNA copy count analysis and subsequent assembly. Reads exceeding this threshold were selected for further investigation.

Sequenced read length distributions were assessed, revealing some reads as short as 100-200 bases (Extended Data Fig. 3c). Considering the long-read nature of the Nanopore sequencing, these short reads are suspected to be contaminants rather than the target DNA fragment. Reads shorter than 250 bases were therefore excluded. A noticeable lack of sequencing yield was observed for the P2 run, where the overall distribution spans much lower frequency compared to the first two rounds of sequencing conducted on P1 and WT preparations (Extended Data Fig. 3c). WT reads primarily exhibited shorter lengths, rendering them unsuitable for non-hybrid de novo assembly (Extended Data Fig. 3d). Based on the initial quality assessment, P1 reads were selected for full genome assembly of P. goldsteinii EVs.

QC metrics indicate varying levels of reliability between P2 and WT samples in distinct analytical settings. For analysis involving read alignments to the reference genome, P2 samples manifest limited reliability due to a high basecalling error rate and a restricted number of sequenced reads. Conversely, in de novo assemblies that rely solely on long reads, WT samples are less reliable due to limited contiguity and coverage.

Notes to Supplementary Table 1

The genomic DNA (gDNA) concentrations were 1072.8 ng for the first mutant extracellular vesicle isolate (P1), 87.75 ng for the second mutant isolate (P2), and 67.65 ng for the wild-type (WT) EV isolate (Supplementary table 1).

Notes to Supplementary Table 2

According to the PycoQC results from Table 2, the P1 and WT runs yielded over six times the number of reads compared to P2, which can be explained by the significantly higher active flowcell channels for the P1 and WT runs. However, the mean read of the WT run was nearly half the mean P2 reads, resulting in a significantly lower number of sequenced bases than the P1 run. This partly explains the significantly low N50 Length in WT.

Notes to Supplementary Table 3 and Extended Data Fig. 4

The Phred quality score (Q) is logarithmically determined by the probability of base-calling errors (P) as $Q = -10 \log_{10}(P)$[106]. Therefore, the mean basecalling error probability can be calculated as Pmean=10−Qmean 10 . Hence, the Phred score of 10, in Supplementary Table 3, corresponds to a basecalling error probability of 0.1, equivalent to 90% accuracy. Similarly, a Phred score 12 corresponds to an error probability of approximately 0.0631. Based on Supplementary Table 3, the basecalling accuracy of the filtered reads for P1, WT, and P2 were approximately 94.8%, 93.4%, and 92.9%, respectively. Additionally, 95% of the P1 reads, 90.3% of the WT reads, and 88.2% of the P2 reads had a basecalling accuracy above 90%.

In Supplementary Extended Data Fig. 4a, the N50 value is delineated on the log-transformed read length distribution. The N50 score is determined by arranging a collection of fragment lengths in descending order and progressively adding the subsequent smallest fragment until the cumulative sum surpasses half of the aggregate sequence length. As presented in Supplementary Table 3 and Extended Data Fig. 4a, the N50 lengths for P1 and P2 are 2010 and 2440 bp, respectively, highlighting that these samples contained longer and more contiguous reads relative to the WT sample, which has an N50 value of 693 bp. It is critical to note that the plots for both WT and P2 samples demonstrate restricted sequence coverage, attributed to shorter gDNA fragments in the WT preparation and a reduced flow cell yield in the P2 sample. In Extended Data Fig. 4b, the Kernel Density Estimate (KDE) plot, plotted on a normalized scale, outlines the read quality distributions for the P1, WT, and P2 samples. The plot indicates that the P1 sample demonstrates a slightly higher level of read quality, with a median score of 14, in comparison to 12.7 for the WT sample and 12.4 for the P2 sample.

Notes to Supplementary Table 4

Supplementary Table 4 provides a comparative summary of experimental and simulated sequences generated using NanoEx-Gen. For each sample, identified by its prefix, both data types exhibit consistent sequence counts, validating the simulation's accuracy in replicating sequence numbers. Notably, sequence lengths exhibit variations due to the error introduction in the simulation, mirroring real-world sequencing profiles. The resemblance between simulated and experimental read profiles underscores the reliability of the NanoEx-Gen simulation.

The mapping efficiency to the reference genome was determined by multiplying the ratio of mapped reads to total sequences by 100. The lower mapping efficiency in the WT sample may be due to the shorter average read length, potential sequence variation, or technical artifacts. The simulated reads exhibited a near-perfect mapping efficiency since they are derived from the reference genome used in the alignment procedure. P1 samples and simulations have the most bases mapped, indicating a larger sequencing depth. The mapping error rate is determined by dividing the number of mismatches by the number of mapped bases (CIGAR). The simulated reads demonstrate a comparable error rate to their experimental counterparts, as the simulation process accounts for the read length and associated error profile. The slightly lower mapping efficiency observed in the simulated counterparts of the WT sample reflects the reduced mapping efficiency observed in the WT sample, which is attributable to shorter mean read length and fragmented gDNA preparation.

Supplementary Files

This is a list of supplementary files associated with this preprint. Click to download.

- tablestatsreport.xlsx
